# Exploring intentions of physician-scientist trainees: factors influencing MD and MD/PhD interest in research careers

**DOI:** 10.1186/s12909-017-0954-8

**Published:** 2017-07-11

**Authors:** Jennifer M. Kwan, Dania Daye, Mary Lou Schmidt, Claudia Morrissey Conlon, Hajwa Kim, Bilwaj Gaonkar, Aimee S. Payne, Megan Riddle, Sharline Madera, Alexander J. Adami, Kate Quinn Winter

**Affiliations:** 1 American Physician Scientists Association, Westford, MA USA; 2 Internal Medicine Physician Scientist Training Program, University of Illinois Chicago, College of Medicine, Chicago, USA; 3 Brigham and Women’s Hospital, Boston, USA; 4 Harvard Medical School, Boston, USA; 5 Pediatrics, University of Illinois Chicago, College of Medicine, Chicago, USA; 6 United States Agency for International Development, Washington, USA; 7 Saving Mothers, Giving Life, Washington, USA; 8 Center for Clinical Translational Sciences, University of Illinois Chicago, College of Medicine, Chicago, USA; 9 University of California, Los Angeles, USA; 10 Dermatology, University of Pennsylvania, Perelman School of Medicine, Philadelphia, USA; 11 University of Washington, Seattle, USA; 12 Medical Scientist Training Program, Weill Cornell Medical College, New York, USA; 13 MD/PhD Program, University of Connecticut Health, Farmington, CT USA; 14 Doctoral Program in Leadership, Creighton University, Omaha, USA

## Abstract

**Background:**

Prior studies have described the career paths of physician-scientist candidates after graduation, but the factors that influence career choices at the candidate stage remain unclear. Additionally, previous work has focused on MD/PhDs, despite many physician-scientists being MDs. This study sought to identify career sector intentions, important factors in career selection, and experienced and predicted obstacles to career success that influence the career choices of MD candidates, MD candidates with research-intense career intentions (MD-RI), and MD/PhD candidates.

**Methods:**

A 70-question survey was administered to students at 5 academic medical centers with Medical Scientist Training Programs (MSTPs) and Clinical and Translational Science Awards (CTSA) from the NIH. Data were analyzed using bivariate or multivariate analyses.

**Results:**

More MD/PhD and MD-RI candidates anticipated or had experienced obstacles related to balancing academic and family responsibilities and to balancing clinical, research, and education responsibilities, whereas more MD candidates indicated experienced and predicted obstacles related to loan repayment. MD/PhD candidates expressed higher interest in basic and translational research compared to MD-RI candidates, who indicated more interest in clinical research. Overall, MD-RI candidates displayed a profile distinct from both MD/PhD and MD candidates.

**Conclusions:**

MD/PhD and MD-RI candidates experience obstacles that influence their intentions to pursue academic medical careers from the earliest training stage, obstacles which differ from those of their MD peers. The differences between the aspirations of and challenges facing MD, MD-RI and MD/PhD candidates present opportunities for training programs to target curricula and support services to ensure the career development of successful physician-scientists.

**Electronic supplementary material:**

The online version of this article (doi:10.1186/s12909-017-0954-8) contains supplementary material, which is available to authorized users.

## Background

Physician-scientists dedicate their careers through research to advancing knowledge of human disease and developing new treatments and preventive measures to improve human health. While few in number, physician-scientists have exerted a substantial impact on medical science, reflected in the outsized majority of Nobel Prizes in Physiology or Medicine that have been awarded to physician-scientists [1]. Physician-scientists are uniquely placed to advance medical science by virtue of their clinical duties. Many significant advances in medicine have arrived by way of a curious observation made by a physician-scientist on the hospital wards [2] that were later expanded and refined in a basic science laboratory. Unsurprisingly, physician-scientists have historically been well-represented in academic medicine. MD/PhD dual degree programs, designed to train physician-scientists, have been particularly successful in this area, with studies of graduates over the last forty years finding that more than three quarters held academic positions and performed research, with well over half serving as principal investigators on NIH grants [3–7].

However, the accolades of the past belie a present crisis facing physician-scientists. The alarm was first sounded in 1979 by James Wyngaarden, who declared the physician-scientist an “endangered species” [8], and subsequent warnings and calls to action have repeatedly pointed to the “vanishing physician-scientist” [1, 9–15]. Unfortunately, despite decades of forewarning and many efforts to reverse the loss of physician-scientists, the picture has grown grim. Attrition rates for new medical school faculty, including MD, MD-PhD, and PhD, are approaching 50% [16–18], and the proportion able to successfully obtain postdoctoral or career development awards (CDAs, e.g. NIH K08 mentored fellowships) has been in decline for many years, with less than 40% of applicants successfully obtaining a CDA [19]. Of those who do successfully receive such awards, approximately one-third never progress to their first independent NIH R01 [13], a difficulty reflected in the ever-rising age at which a junior physician-scientist receives his or her first independent research grant [17, 19, 20]. Unsurprisingly, physician-scientists are making up an increasingly smaller portion of the overall pool of NIH-funded researchers [11, 20–22] In short, the physician-scientist pathway has become a very leaky pipeline [1, 13], with many candidates lost at every stage of training.

In the face of this crisis, many groups have endeavored to determine the factors that underlie the disappearance of physician-scientists. Multiple efforts have been made to identify the reasons behind high attrition rates for junior medical school faculty [5, 17, 18, 23–28], as well as the struggles facing even more senior faculty [9–11]. Others have studied postgraduates, those undergoing residency and fellowship training, in the hopes that assisting trainees earlier in their careers may prevent future attrition [5, 12, 26, 29–31]. Importantly, attrition exists even at the initial stages of the physician-scientist pipeline, with 10–15% of MD/PhD program trainees failing to complete their studies [32]. Additionally, one means to increase the number of physician-scientists would be to increase the number of trainees embarking on this pathway, increasing the likelihood that a larger group will survive the leaky pipeline [32] and embark on a research career. Recognizing this, a number of surveys of MD/PhD programs, where many physician-scientists begin their training, were conducted [4, 6, 33–40]. These studies have provided valuable insights into the career choices of young physician scientists and some of the factors behind them, but our picture remains incomplete. Importantly, no work has been performed at the predoctoral level to evaluate the influence of factors such as a desire for work-life balance and concerns about raising a family on the decision to enter an academic and research career. Identification of such factors early in training may provide insight into the reasons why trainees choose not to continue on the physician-scientist pathway, whether in the medical school stage or later in their careers, and could help training programs provide an environment which encourages these individuals to continue in research careers. Furthermore, relatively little work has been done to identify and study MD trainees interested in performing research [41–44], historically a key population of physician-scientists but one which has remained disturbingly flat in recent years despite rising enrollment in MD programs [20, 45]. To address these deficits, our study aimed to assess the characteristics of physician-scientist trainees, including MD and MD/PhD candidates, in terms of planned specialty, career sector intentions, and unique to this study, the influence of factors such as work-life balance and desire to start a family on their career decisions.

## Methods

### Data collection

A 70-item survey was designed with feedback from a survey design team at the University of Illinois at Chicago. The survey instrument designed for this study utilized with permission four items from Quinn [46], which were single-item measures with face validity (i.e., selecting the area of intended career sector and intention, most important factor in career selection, etc.) and therefore, internal reliability scores are not calculable. The majority of measures in the instrument are single-item and were developed specifically for this study to permit exploration of differences in the specific aspirations and experiences reported by various respondent characteristics. Assessment of the face validity of items was conducted by experts in the field. Data were collected using an online survey tool (SurveyMonkey, www.surveymonkey.com). The study was reviewed and exempted by the Institutional Review Board at five major US institutions: University of Illinois at Chicago, University of Pennsylvania, Weill Cornell Medical College, Northwestern University, and University of Chicago. The survey was sent in September 2011 via e-mail to all MD and MD/PhD students at these universities through student listservs and the institutional representatives of the American Physician Scientists Association (APSA). Participants consented on the 1st page of the surveymonkey link prior to starting the survey. Participants also had an option to enter an institutional email address for a $50 Amazon gift certificate to be chosen at random. Data collection ended December 2012. E-mail addresses were kept separate from survey responses to maintain confidentiality of responses.

### Statistical analysis

For sample size of 2757 with confidence interval of 95% and margin of error of 3%, we aimed to get above 27% response rate, which we achieved with a response of 1103, a response rate of 40%. All submitted surveys were included, even if they were not fully completed. MD/PhD students were identified through how they paid for medical school as being sponsored by an MD/PhD program. MD candidates interested in research-intensive careers (MD-RI) were identified by their career intentions of wanting a research to clinical duty ratio of 50% or greater. Half time or greater devotion to research efforts was selected to include trainees intending to become surgeon-scientists in the analysis. Surgeon-scientists are permitted lower full-time effort devoted to research on mentored NIH career development awards [47]. Survey results were analyzed to identify significant differences between the MD and MD/PhD candidates, as well as the factors that influence their intentions. Chi squared tests were used to measure the significance of associations between categorical variables, such as whether MD and MD/PhD candidates differed in their responses to specialization interests. Where data violated minimum expected cell counts, Fisher’s exact test was performed. Logistic regression was used to determine the unique influence of each predictor variable on the intention to go into academic medicine after residency, controlling for other variables in the model, such as MD or MD/PhD candidate status. All tests were performed using SPSS. All tests of significance were 2-sided and *p* < .05 was considered significant.

## Results

### Respondent characteristics

The survey yielded 1103 responses, a 40% response rate from the 2757 MD and MD/PhD candidates (50% of surveyed MD/PhD candidates and 32% of surveyed MD candidates) who received the introductory email to this pilot study, representing students from all training stages. MD/PhD candidates were 20% (*n* = 226), MD-RI was 16.6% (*n* = 184) and MD candidates were 62.8% (*n* = 693) of respondents. Table 1 shows the descriptive statistics for gender, training stage, race, ethnicity, marital status, and parental status by MD, MD RI or MD/PhD candidate status, with whether any difference by candidate status is statistically significant. Roughly equal numbers of males (*n* = 511) and females (*n* = 548) responded and there was no significant difference by gender of MD/PhD or MD candidate respondents. Women comprise 47.6% of the MD/PhD respondents, 53.8% of MD RI and 52.6% of the MD respondents. While the proportion of women MD respondents is comparable to the national average [45], the proportion of women MD/PhD respondents is somewhat higher than the national average of 37.9% as of 2012. [48] There was a higher proportion of M1 students represented (27.5%) compared to M2 (20.2%), M3 (18.9%) and M4 (20.2%), although statistical analysis was not performed due to minimum expected cell count violation. Further, the categories G1-G5 or more represent MD/PhD students in their PhD training years and therefore it was expected that a higher percentage of MD/PhD students than MD students would indicate being in those stages. MD candidates can be in the G1-G5 categories due to taking a year out to obtain a Master’s degree or taking a break to pursue a separate PhD before returning to clinical training. A greater proportion of MD candidates (10.1%) than MD/PhD candidates (3.5%) indicated being Hispanic (*p =* 0.001). Higher proportions of MD/PhD respondents (32.6%) than MD respondents (19.1%) indicated being married/partnered (*p =* 0.001) and having a child or children (6.3% and 2.9%, respectively; *p =* 0.028). 45.9% of respondents indicated that their mother has an advanced degree. There are significant differences (*p* < .05) in the distributions of advanced degrees of mother between the three medical candidate groups for MD or DO and for DVM. For the 9.2% of respondents who indicated that their mother is working in an area of medicine, no significant differences in the area of medicine (academia, private practice, consulting, or industry) were identified. 63.7% of respondents indicated their father had an advanced degree, with the only significant differences in distribution between groups in where the father has a MD or DO or a DVM. Finally, 21.6% of respondents indicated that their father works in an area of medicine, with the only difference between groups being when the father works in academic medicine, for example more MD PhD and MD RI have fathers working in academia than MD trainees.

**Table 1:** Demographic Characteristics of Respondents by MD, MD-RI, or MD/PhD

Demographic	Total, n (%)	MD/PhD, n (%)	MD-RI, n (%)	MD, n (%)	*P* value
Gender					>.05
Female	548 (51.7%)	107 (47.6%)	99 (53.8%)	342 (52.6%)	
Male	511 (48.3%)	118 (52.4%)	85 (46.2%)	308 (47.4%)	
TOTAL	1059 (100%)	225 (100%)	184 (100%)	650 (100%)	
Training stage					**<.001**
Medical School Year 1	285 (27.5%)	24 (11.2%)	55 (31.1%)	206 (32.0%)	
Medical School Year 2	209 (20.2%)	26 (12.1%)	40 (22.6%)	143 (22.2%)	
Medical School Year 3	196 (18.9%)	22 (10.2%)	38 (21.5%)	136 (21.1%)	
Medical School Year 4	209 (20.2%)	21 (9.8%)	43 (24.3%)	145 (22.5%)	
Graduate School Year 1	36 (3.5%)	25 (11.6%)	1 (0.6%)	10 (1.6%)	
Graduate School Year 2	37 (3.6%)	35 (16.3%)	0 (−-)	2 (0.3%)	
Graduate School Year 3	28 (2.7%)	27 (12.6%)	0 (−-)	1 (0.2%)	
Graduate School Year 4	22 (2.1%)	21 (9.8%)	0 (−-)	1 (0.2%)	
Graduate School Year 5 or more	14 (1.4%)	14 (6.5%)	0 (−-)	0 (−-)	
TOTAL	1036 (100%)	215 (100%)	177 (100%)	644 (100%)	
Race					>.05
White	633 (60.5%)	138 (61.1%)	101 (55.5%)	394 (61.8%)	
Black or African American	71 (6.8%)	15 (6.6%)	15 (8.2%)	41 (6.4%)	
American Indian or Alaska Native	4 (0.4%)	0 (−-)	0 (−-)	4 (0.6%)	
Asian or Pacific Islander	228 (21.8%)	52 (23.0%)	42 (23.1%)	134 (21.0%)	
Multi-racial or Other	78 (7.5%)	15 (6.6%)	17(9.3%)	46 (7.2%)	
Prefer not to answer	32 (3.1%)	6 (2.7%)	7 (3.8%)	19 (3.0%)	
TOTAL	1046 (100%)	226 (100%)	182 (100%)	638 (100%)	
Ethnicity					**<.01**
Hispanic	97 (9.2%)	8 (3.5%)	24 (13.0%)	65 (10.1%)	
Not Hispanic	958 (90.8%)	218 (96.5%)	160 (87.0%)	580 (89.9%)	
TOTAL	1055 (100%)	226 (100%)	184 (100%)	645 (100%)	
Marital Status					**<.001**
Is married/partnered	226 (22.0%)	73 (32.6%)	33 (17.9%)	120 (19.4%)	
Is NOT married/partnered	799 (78.0%)	151 (67.4%)	151 (82.1%)	497 (80.6%)	
TOTAL	1025 (100%)	224 (100%)	184 (100%)	617 (100%)	
Parental Status					>.05
Has a child/children (of 1025)	37 (3.6%)	14 (6.3%)	5 (2.7%)	18 (2.9%)	
Does NOT have a child/children	988 (96.4%)	210 (93.8%)	179 (97.3%)	599 (97.1%)	
TOTAL	1025 (100%)	224 (100%)	184 (100%)	617 (100%)	
	Total, n (% of 1103)	MD/PhD, n (% of 226)	MD-RI, n (% of 184)	MD, n (% of 693)	*P* value
Advanced degree of mother^a^					
MD or DO	172 (15.6%)	46 (20.4%)	37 (20.1%)	89 (12.8%)	**<.01**
DDS	4 (0.4%)	1 (0.4%)	1 (0.5%)	2 (0.3%)	>.05^b^
PhD	32 (2.9%)	7 (3.1%)	5 (2.7%)	20 (2.9%)	>.05
JD	7 (0.6%)	0 (−-)	2 (1.1%)	5 (0.7%)	>.05^b^
DVM	203 (18.4%)	49 (21.7%)	42 (22.8%)	112 (16.2%)	**<.05**
Master’s	88 (8.0%)	11 (4.9%)	12 (6.5%)	65 (9.4%)	>.05
Area of medicine mother works in^a^					
Academia	35 (3.2%)	6 (2.7%)	10 (5.4%)	19 (2.7%)	>.05
Private practice	58 (5.3%)	16 (7.1%)	12 (6.5%)	30 (4.3%)	>.05
Consulting	4 (0.4%)	1 (0.4%)	1 (0.5%)	2 (0.3%)	>.05^b^
Industry	3 (0.3%)	1 (0.4%)	0 (−-)	2 (0.3%)	>.05^b^
Advanced degree of father^a^					
MD or DO	341 (30.9%)	84 (37.2%)	68 (37.0%)	189 (27.3%)	**<.01**
DDS	14 (1.3%)	3 (1.3%)	1 (0.5%)	10 (1.4%)	>.05
PhD	89 (8.1%)	15 (6.6%)	10 (5.4%)	64 (9.2%)	>.05
JD	12 (1.1%)	1 (0.4%)	2 (1.1%)	9 (1.3%)	>.05
DVM	188 (17.0%)	53 (23.5%)	33 (17.9%)	102 (14.7%)	**<.05**
Master’s	59 (5.3%)	6 (2.7%)	13 (7.1%)	40 (5.8%)	>.05
Area of medicine father works in^a^					
Academia	86 (7.8%)	21 (9.3%)	23 (12.5%)	42 (6.1%)	**<.05**
Private practice	129 (11.7%)	21 (9.3%)	23 (12.5%)	85 (12.3%)	>.05
Consulting	11 (1.0%)	1 (0.4%)	1 (0.5%)	9 (1.3%)	>.05^b^
Industry	12 (1.1%)	2 (0.9%)	2 (1.1%)	8 (1.2%)	>.05^b^
How primarily paid for medical school^a^					
MD-PhD or DO-PhD sponsored	226 (20.5%)	226 (100%)	0 (−-)	0 (−-)	**<.001**
Scholarships	327 (29.6%)	7 (3.1%)	81 (44.0%)	239 (34.5%)	**<.001**
Grants	185 (16.8%)	16 (7.1%)	42 (22.8%)	127 (18.3%)	**<.001**
Loans	636 (57.7%)	17 (7.5%)	133 (72.3%)	486 (70.1%)	**<.001**
National Service	15 (1.4%)	0 (−-)	3 (1.6%)	12 (1.7%)	>.05^b^
Personal Savings	124 (11.2%)	5 (2.2%)	24 (13.0%)	95 (13.7%)	**<.001**
Family/partner Support	302 (27.4%)	5 (2.2%)	75 (40.8%)	222 (32.0%)	**<.001**
Work	54 (4.9%)	1 (0.4%)	15 (8.2%)	38 (5.5%)	**<.01**
Work Study	7 (0.6%)	1 (0.4%)	2 (1.1%)	4 (0.6%)	>.05^b^

^a^Respondents could select all applicable choices, will not sum to 100%

^b^Fisher’s Exact calculated due to minimum cell count violations

bolded are statistically significant

The final area of respondent characteristics is how they primarily paid for medical school. As expected, significant differences in sources of financial support were observed between MD-PhD and other groups, with the exception of national service and work study. Age was requested, but too few respondents provided their age for it to be used for analysis (Table 1).

### Career trajectories and specialty interests

There is considerable interest in understanding the career trajectory of MD/PhD students and how these compare to their MD-RI and MD counterparts. Our study found that 94.2% of MD/PhD students and 88.0% of MD-RI students intend a career in academics, compared to 67.8% of MD students (*p <* 0.001). Some 25.7% of MD/PhD respondents express interest in a career in industry, versus 9.8% of MD-RI students and 9.7% of MD students (*p <* 0.001). MD/PhD respondents were more likely than their MD-RI and MD counterparts to pursue a career in consulting (22.1% vs 15.2% and 12.7%, respectively; *p* < 0.01). Conversely, 54.5% of MD students and 39.1% of MD-RI students are planning a career in private practice, compared to 17.7% of MD/PhD students (*p <* 0.001). MD/PhD respondents express a higher interest than do MD-RI and MD respondents in basic research (59.3% vs 14.7% and 3.9%, respectively; *p <* 0.001) and translational research (68.1% vs. 39.1% and 14.0%, respectively; *p <* 0.001). MD-RI respondents express a greater interest in clinical research than their MD/PhD and MD counterparts (67.4% vs. 37.2% and 47%, respectively; *p* < 0.001). MD-RI respondents are more likely than MD/PhD and MD respondents to plan to incorporate advocacy (34.2% vs. 19.5% and 29%, respectively; *p* < 0.01) and administration into their career plans (31.0% vs. 24.3% and 20.2%; *p <* 0.001). Table 2 details distributions by sector interest and career intention by MD, MD-RI, or MD/PhD status.

**Table 2:** Career sector and specialty intentions of MD, MD-RI or MD/PhD trainees

	Total, n (% of 1103)	MD/PhD, n (% of 226)	MD-RI, n (% of 184)	MD, n (% of 693)	*P* value
Sector^a^					
Academia	845 (76.6%)	213 (94.2%)	162 (88.0%)	470 (67.8%)	**<.001**
Private practice	490 (44.4%)	40 (17.7%)	72 (39.1%)	378 (54.5%)	**<.001**
Consulting	166 (15.0%)	50 (22.1%)	28 (15.2%)	88 (12.7%)	**<.01**
Industry	143 (13.0%)	58 (25.7%)	18 (9.8%)	67 (9.7%)	**<.001**
Career Intention^a^					
Clinical duties	870 (78.9%)	172 (76.1%)	151 (82.1%)	547 (78.9%)	>.05
Education	660 (59.8%)	143 (63.3%)	128 (69.6%)	389 (56.1%)	**<.01**
Clinical research	534 (48.4%)	84 (37.2%)	124 (67.4%)	326 (47.0%)	**<.001**
Translational research	323 (29.3%)	154 (68.1%)	72 (39.1%)	97 (14.0%)	**<.001**
Advocacy	308 (27.9%)	44 (19.5%)	63 (34.2%)	201 (29.0%)	**<.01**
Administration	252 (22.8%)	55 (24.3%)	57 (31.0%)	140 (20.2%)	**<.01**
Basic research	188 (17.0%)	134 (59.3%)	27 (14.7%)	27 (3.9%)	**<.001**
Specialty^b^					
Allergy and Immunology	14 (1.3%)	8 (3.5%)	4 (2.2%)	2 (.03%)	**<.001** ^**c**^
Anesthesiology	77 (7.0%)	16 (7.1%)	9 (4.9%)	52 (7.5%)	>.05
Colon and Rectal Surgery	2 (0.2%)	0 (−-)	1 (0.5%)	1 (0.1%)	>.05^c^
Dermatology	68 (6.2%)	19 (8.4%)	12 (6.5%)	37 (5.3%)	>.05
Emergency Medicine	160 (14.5%)	15 (6.6%)	31 (16.8%)	114 (16.5%)	**<.001**
Family Medicine	96 (8.7%)	2 (0.9%)	14 (7.6%)	80 (11.5%)	**<.001**
Internal Medicine	435 (39.4%)	107 (47.3%)	82 (44.6%)	246 (35.5%)	**<.01**
Medical Genetics	13 (1.2%)	9 (4.0%)	1 (0.5%)	3 (0.4%)	**<.001**
Neurological Surgery	17 (1.5%)	0 (−-)	5 (2.7%)	12 (1.7%)	**<.05** ^**c**^
Neurology	88 (8.0%)	41 (18.1%)	13 (7.1%)	34 (4.9%)	**<.001**
Nuclear Medicine	1 (0.1%)	0 (−-)	0 (−-)	1 (0.1%)	>.05^c^
Obstetrics and Gynecology	73 (6.6%)	7 (3.1%)	15 (8.2%)	51 (7.4%)	>.05
Ophthalmology	56 (5.1%)	14 (6.2%)	10 (5.4%)	32 (4.6%)	>.05
Orthopaedic Surgery	61 (5.5%)	5 (2.2%)	12 (6.5%)	44 (6.3%)	**<.05**
Otolaryngology	33 (3.0%)	1 (0.4%)	7 (3.8%)	25 (3.6)	**<.05**
Pathology	56 (5.1%)	45 (19.9%)	2 (1.1%)	9 (1.3%)	**<.001**
Pediatrics	220 (19.9%)	46 (20.4%)	33 (17.9%)	141 (20.3%)	>.05
Physical Medicine and Rehabilitation	11 (1.0%)	1 (0.4%)	2 (1.1%)	8 (1.2%)	>.05^c^
Plastic Surgery	31 (2.8%)	5 (2.2%)	6 (3.3%)	20 (2.9%)	>.05
Preventative Medicine	16 (1.5%)	0 (−-)	6 (3.3%)	10 (1.4%)	**<.05** ^**c**^
Psychiatry	69 (6.3%)	20 (8.8%)	14 (7.6%)	35 (5.1%)	>.05
Radiation Oncology	36 (3.3%)	15 (6.6%)	11 (6.0%)	10 (1.4%)	**<.001**
Radiology	77 (7.0%)	19 (8.4%)	20 (10.9%)	38 (5.5%)	**<.05**
Surgery	120 (10.9%)	8 (3.5%)	21 (11.4%)	91 (13.1%)	**<.001**
Thoracic Surgery	14 (1.3%)	0 (−-)	1 (0.5%)	13 (1.9%)	>.05^c^
Urology	29 (2.6%)	5 (2.2%)	7 (3.8%)	17 (2.5%)	>.05

^﻿a^Respondents could select all applicable choices, will not sum to 100%

^b^Respondents could select up to TWO choices, will not sum to 100%

^c^Fisher's Exact calculated due to minimum cell count violations

bolded are statistically significant

Specialty plans differ between MD, MD-RI, and MD/PhD students. Overall, MD and MD-RI respondents were more likely than their MD/PhD counterparts to consider surgical specialties, such as orthopaedic surgery (6.3% and 6.5% vs. 2.2%, respectively; *p* < 0.05), otolaryngology (3.6% and 3.8% vs. 0.4%, respectively; *p* < 0.05), and surgery (13.1% and 11.4% vs. 3.5%, respectively; *p* < 0.001). Similarly, acute care specialties such as emergency medicine attract fewer MD/PhD candidates (6.6%) compared to MD candidates (16.5%) and MD-RI candidates (16.8%; *p* < 0.001). Primary care specialties, such as internal medicine, attract more MD/PhD (47.3%) and MD-RI (44.6%) candidates compared to MD candidates (35.5%; *p* < 0.01), while family medicine attracts more MD candidates (11.5%) and MD-RI candidates (7.6%) compared to MD/PhD candidates (0.9%; *p* < 0.001). Certain diagnostic specialties attract more MD/PhD candidates compared to MD and MD-RI candidates, including medical genetics (4.0% vs. 0.4% and 0.5%, respectively; *p* < 0.001), neurology (18.1% vs. 4.9% and 7.1%, respectively; *p* < 0.001), and pathology (19.9% vs. 1.3% and 1.1%, respectively; *p* < 0.001). Other diagnostic specialties attract more MD/PhD and MD-RI candidates compared to MD candidates, including radiology (8.4% and 10.9% vs. 5.5%, respectively; *p* < 0.05) and radiation oncology (6.6% and 6.0% vs. 1.4%, respectively; *p* < 0.001). Table 2 provides the complete overview of distributions by specialty and MD or MD/PhD status.

### Current and foreseen career obstacles

Distributions of responses across the three candidate groups differ significantly on the foreseeable non-work-related responsibilities during and after residency (Table 3). Raising children was the most frequently indicated item for all groups in both time frames (70.8%, 55.4%, and 50.8% during residency for MD/PhD, MD-RI, and MD candidates, respectively *p* < .001; and 85.0%, 91.3%, and 80.7% after residency, *p* < .01).

**Table 3:** Obstacles and important factors influencing careers of MD, MD-RI and MD PhD trainees

Foreseeable non-work-related responsibilities DURING residency					
	Total, n (% of 1103)	MD/PhD, n (% of 226)	MD-RI, n (% of 184)	MD, n (% of 693)	*P* value
Raising children	614 (55.7%)	160 (70.8%)	102 (55.4%)	352 (50.8%)	**<.001**
Taking care of elderly parents	187 (17.0%)	52 (23.0%)	32 (17.4%)	103 (14.9%)	**<.05**
Being caretaker to others	130 (11.8%)	17 (7.5%)	36 (19.6%)	77 (11.1%)	**<.001**
Financial support of others	267 (24.2%)	44 (19.5%)	60 (32.6%)	163 (23.5%)	**<.01**
Foreseeable non-work-related responsibilities AFTER residency^a^					
Raising children	919 (83.3%)	192 (85.0%)	168 (91.3%)	559 (80.7%)	**<.01**
Taking care of elderly parents	678 (61.5%)	160 (70.8%)	131 (71.2%)	387 (55.8%)	**<.001**
Being caretaker to others	322 (29.2%)	42 (18.6%)	77 (41.8%)	203 (29.3%)	**<.001**
Financial support of others	549 (49.8%)	99 (43.8%)	109 (59.2%)	341 (49.2%)	**<.01**
Experienced Obstacles^a^					
Balancing family and work responsibilities	396 (35.9%)	85 (37.6%)	73 (39.7%)	238 (34.3%)	>.05
Balance clinical, research, & education responsibilities	240 (21.8%)	70 (31.0%)	56 (30.4%)	114 (16.5%)	**<.001**
Loan repayment	172 (15.6%)	1 (0.4%)	33 (17.9%)	138 (19.9%)	**<.001**
Lack of opportunity/funding	143 (13.0%)	28 (12.4%)	38 (20.7%)	77 (11.1%)	**<.01**
Satisfactory professional development	95 (8.6%)	23 (10.2%)	14 (7.6%)	58 (8.4%)	>.05
Under-compensation	70 (6.3%)	27 (11.9%)	9 (4.9%)	34 (4.9%)	**<.001**
Discrimination/biases (gender/ethnicity)	67 (6.1%)	14 (6.2%)	14 (7.6%)	39 (5.6%)	>.05
Not finding position in desired location	62 (5.6%)	10 (4.4%)	17 (9.2%)	35 (5.1%)	>.05
Sexual harassment	10 (0.9%)	3 (1.3%)	2 (1.1%)	5 (0.7%)	>.05^c^
Malpractice/lawsuit	4 (0.4%)	0 (−-)	1 (0.5%)	3 (0.4%)	>.05^c^
Predicted Obstacles^a^					
Balancing family and work responsibilities	857 (77.7%)	189 (83.6%)	148 (80.4%)	520 (75.0%)	**<.05**
Balance clinical, research, & education responsibilities	549 (49.8%)	186 (82.3%)	115 (62.5%)	248 (35.8%)	**<.001**
Not finding position in desired location	445 (40.3%)	119 (52.7%)	77 (41.8%)	249 (35.9%)	**<.001**
Loan repayment	396 (35.9%)	10 (4.4%)	81 (44.0%)	305 (44.0%)	**<.001**
Under-compensation	301 (27.3%)	50 (22.1%)	66 (35.9%)	185 (26.7%)	**<.01**
Lack of opportunity/funding	279 (25.3%)	123 (54.4%)	60 (32.6%)	96 (13.9%)	**<.001**
Malpractice/lawsuit	267 (24.2%)	17 (7.5%)	54 (29.3%)	196 (28.3%)	**<.001**
Satisfactory professional development	264 (23.9%)	68 (30.1%)	53 (28.8%)	143 (20.6%)	**<.01**
Discrimination/biases (gender/ethnicity)	132 (12.0%)	22 (9.7%)	27 (14.7%)	83 (12.0%)	>.05
Sexual harassment	14 (1.3%)	3 (1.3%)	4 (2.2%)	7 (1.0%)	>.05^d^
Career Intention Time Allocation: Research/Clinical Ratios					
	Total, n (% of 1103)	MD/PhD, n (% of 226)	MD-RI, n (% of 184)	MD, n (% of 693)	*P* value
100/0	18 (1.6%)	8 (3.5%)	10 (5.4%)	0 (−-)	**<.001** ^c^
75/25	152 (13.8%)	111 (49.1%)	41 (22.3%)	0 (−-)	**<.001**
50/50	209 (18.9%)	75 (33.2%)	134 (72.8%)	0 (−-)	**<.001**
25/75	529 (48.0%)	34 (15.0%)	17 (9.2%)	478 (69.0%)	**<.001**
0/100	138 (12.5%)	2 (0.9%)	1 (0.5%)	135 (19.5%)	**<.001**
Most important factors in career selection^b^					
Ability to balance work & personal life	746 (67.6%)	161 (71.2%)	130 (70.7%)	455 (65.7%)	>.05
Opportunities for patient care	708 (64.2%)	116 (51.3%)	131 (71.2%)	461 (66.5%)	**<.001**
Financial security	375 (34.0%)	82 (36.3%)	69 (37.5%)	224 (32.3%)	>.05
Opportunities to teach	333 (30.2%)	53 (23.5%)	56 (30.4%)	224 (32.3%)	<.05
Opportunities for research	298 (27.0%)	158 (69.9%)	77 (41.8%)	63 (9.1%)	**<.001**
Opportunities for community service	214 (19.4%)	15 (6.6%)	31 (16.8%)	168 (24.2%)	**<.001**
Opportunities for international work	160 (14.5%)	21 (9.3%)	36 (19.6%)	103 (14.9%)	**<.05**
Autonomy	157 (14.2%)	43 (19.0%)	21 (11.4%)	93 (13.4%)	>.05
Opportunities for student interactions	119 (10.8%)	23 (10.2%)	21 (11.4%)	75 (10.8%)	>.05
Prestige	74 (6.7%)	17 (7.5%)	17 (9.2%)	40 (5.8%)	>.05
Opportunities for travel	73 (6.6%)	17 (7.5%)	10 (5.4%)	46 (6.6%)	>.05
Opportunities for local work	33 (3.0%)	1 (0.4%)	6 (3.3%)	26 (3.8%)	**<.05**
Opportunities for national work	21 (1.9%)	2 (0.9%)	3 (1.6%)	16 (2.3%)	>.05^c^

^﻿a^Respondents could select all applicable choices, will not sum to 100%

^b^Respondents could select up to THREE choices, will not sum to 100%

^c^Fisher's Exact calculated due to minimum cell count violations

bolded are statistically significant

In assessing the obstacles that have hindered their career advancement to date, work-life balance was identified as the top concern for MD/PhD, MD-RI, and MD candidates (37.6%, 39.7% and 34.3% respectively). MD/PhD and MD-RI respondents express more concern in balancing research, clinical, and education responsibilities compared to MD respondents (31.0% and 30.4% vs. 16.5%) (*p <* 0.001). More MD-RI and MD respondents (17.9% and 19.9%) than MD/PhD respondents (0.4%) cite loan repayment as a current obstacle (*p <* 0.001). A higher percentage of MD-RI students (20.7%) cited lack of opportunity/funding as a current obstacle compared to only 12.4% of MD/PhD students and 11.1% of MD students (*p <* 0.01). Conversely, 11.9% of MD/PhD students cited under-compensation as a current obstacle compared to only 4.9% of MD and MD-RI respondents (*p <* 0.001). No statistically significant differences are observed between MD, MD-RI, and MD/PhD respondents for citing discrimination bias, sexual harassment, not finding position in desired location, malpractice, or unsatisfactory professional development as an experienced obstacle. Table 2 includes distributions of all experienced and predicted career obstacles by MD, research-oriented MD or MD/PhD status.

An examination of the obstacles that candidates expect to play a major role in their future careers reveals a number of differences between the three groups of respondents. All three groups identify work-life balance as the top predicted obstacle in their careers, but more MD/PhD candidates (83.6%) and MD-RI candidates (80.4%) than MD candidates (75%) cite work-life balance as a future obstacle they are likely to face (*p <* 0.05). Similarly, a higher percentage of MD/PhD candidates (82.3%) and MD-RI candidates (62.5%) than MD candidates (35.8%) cite balancing clinical, research, and teaching as a predicted career obstacle (*p <* 0.001). More MD/PhD and MD-RI candidates foresee lack of opportunity and funding as well as lack of positions in a desired location as a greater hindrance to their career success compared to MD candidates (*p <* 0.001 for both variables). Additionally, more MD/PhD candidates (30.1%) and MD-RI candidates (28.8%) than MD candidates (20.6%) cite lack of satisfactory professional development as a projected future obstacle (*p < 0.01*). A higher percentage of MD candidates and MD-RI candidates than MD/PhD candidates foresee loan repayment and malpractice as future hindrances in their careers (*p <* 0.001 for both variables). Under-compensation is identified as a predicted obstacle to career success by 35.9% of MD-RI candidates compared to only 22.1% of MD/PhD candidates and 26.7% of MD candidates (*p < 0.01*). No statistically significant differences were observed among the three groups for citing discrimination/bias and sexual harassment as a predicted future obstacle (Table 3).

### Intended time allocation between research and clinical activities

MD and MD/PhD respondents differ in their intended allocation of work-time between research and clinical activities. Specifically, 49.1% of MD/PhD respondents, compared to 4.7% of MD respondents, indicate intention to devote 75% of their work-time to research activities and 25% to clinical activities (*p* < 0.001). Conversely, 56.4% of MD respondents, compared to 15.0% of MD/PhD respondents, plan to commit 75% of their work-time to clinical activities and 25% to research activities (*p* < 0.001). Overall, 85.8% of MD/PhD respondents plan to spend 50% or more time on research, compared to 21.1% of MD respondents.

### Most important factors in choosing a career

The most frequently identified top-three factor in career selection is the ability to balance work and personal life, having been selected by 67.6% of all respondents. However, while this factor was the highest selected by both the MD/PhD and the MD respondents, it was the second highest selected by the MD-RI respondents (after opportunities for patient care). Respondents differ significantly in 5 of the 13 identified factors in choosing a career (Table 2). Higher percentages of MD-RI and MD respondents than MD/PhD respondents select as a top-three important factor in career selection: “opportunities for patient care” (66.5% and 71.2% vs. 51.3%; *p* < 0.001), “opportunities for community service” (16.8% and 24.2% vs. 6.6%; *p* < 0.001), “opportunities for international work” (19.6% and 14.9% vs. 9.3%; *p* < 0.05), and “opportunities for local work” (3.3% and 3.8% vs. 0.4%; *p* < 0.05). Conversely, higher percentages of MD/PhD and MD-RI respondents than MD respondents select “opportunities for research” (69.9% and 41.8% vs. 9.1%; *p* < 0.001) (Table 3).

### Feasibility of research intense careers

More MD/PhD respondents (51.2%) than MD-RI (17.9%) and MD respondents (16.1%) view research intense careers as “highly difficult” in surgical specialties (*p* < 0.001). Likewise, in acute care specialties, 64.3% of MD/PhD respondents indicate that research intense careers would be “difficult,” “highly difficult,” or “impossible,” compared to 48.9% of MD-RI and 54.2% of MD respondents (*p* < 0.001). Consistent with this, fewer MD/PhD (35.8%) than MD-RI (51.1%) and MD (45.7%) view acute care specialties as feasible or highly feasible (*p* < 0.0001) (Table 4).

**Table 4:** Perceptions of Feasibility and Mentoring by MD, MD-RI or MD/PhD

	Total, n (%)	MD/PhD, n (%)	MD-RI, n (%)	MD, n (%)	*P* value
How feasible is a research intense career in acute care medicine specialties?					**< 0.001**
Highly feasible	97 (9.6%)	25 (11.6%)	25 (13.6%)	47 (7.7%)	
Feasible	352 (35.0%)	52 (24.2%)	69 (37.5%)	231 (38.0%)	
Difficult	384 (38.1%)	81 (37.7%)	65 (35.3%)	238 (39.1%)	
Highly difficult	159 (15.8%)	53 (24.7%)	22 (12.0%)	84 (13.8%)	
Impossible	15 (1.5%)	4 (1.9%)	3 (1.6%)	8 (1.3%)	
TOTAL	1007 (100%)	215 (100%)	184 (100%)	608 (100%)	
How feasible is a research intense career in surgical specialties?					**< 0.001**
Highly feasible	73 (7.2%)	2 (0.9%)	21 (11.4%)	50 (8.2%)	
Feasible	309 (30.7%)	29 (13.5%)	65 (35.3%)	215 (35.3%)	
Difficult	347 (34.4%)	59 (27.4%)	62 (33.7%)	226 (37.1%)	
Highly difficult	241 (23.9%)	110 (51.2%)	33 (17.9%)	98 (16.1%)	
Impossible	38 (3.8%)	15 (7.0%)	3 (1.6%)	20 (3.3%)	
TOTAL	1008 (100%)	215 (100%)	184 (100%)	609 (100%)	
Can you currently identify a mentor(s) who has helped you progress toward &/or achieve your career goals?					**< 0.001**
Yes	732 (73.6%)	196 (89.5%)	134 (74.4%)	402 (67.4%)	
No	263 (26.4%)	23 (10.5%)	46 (25.6%)	194 (32.6%)	
TOTAL	995 (100%)	219 (100%)	180 (100%)	596 (100%)	
How important has mentorship been in your training thus far?					**<.001** ^a^
Very important	328 (44.9%)	118 (60.5%)	67 (50.0%)	143 (35.6%)	
Somewhat important	361 (49.4%)	73 (37.4%)	65 (48.5%)	223 (55.5%)	
Not very important	40 (5.5%)	4 (2.1%)	2 (1.5%)	34 (8.5%)	
Not at all important	2 (0.3%)	0 (−-)	0 (−-)	2 (0.5%)	
TOTAL	731 (100%)	195 (100%)	134 (100%)	402 (100%)	
How much importance is given to talents/accomplishments when recruiting applicants for jobs and/or positions in science and medicine?					>.05
A great deal of importance	330 (33.1%)	89 (40.8%)	61 (33.7%)	180 (30.2%)	
A lot of importance	487 (48.9%)	98 (45.0%)	82 (45.3%)	307 (51.4%)	
Moderate amount of importance	162 (16.3%)	29 (13.3%)	33 (18.2%)	100 (16.8%)	
Little importance	14 (1.4%)	2 (0.9%)	3 (1.7%)	9 (1.5%)	
None at all	3 (0.3%)	0 (−-)	2 (1.1%)	1 (0.2%)	
TOTAL	996 (100%)	218 (100%)	181 (100%)	597 (100%)	
How much importance is given to connections/networking when recruiting applicants for jobs and/or positions in science and medicine?					>.05
A great deal of importance	301 (30.2%)	73 (33.3%)	59 (32.8%)	169 (28.3%)	
A lot of importance	388 (39.0%)	81 (37.0%)	71 (39.4%)	236 (39.5%)	
Moderate amount of importance	267 (26.8%)	54 (24.7%)	41 (22.8%)	172 (28.8%)	
Little importance	40 (4.0%)	11 (5.0%)	9 (5.0%)	20 (3.4%)	
TOTAL	996 (100%)	219 (100%)	180 (100%)	597 (100%)	

^a^ Fisher’s Exact calculated due to minimum cell count violations

bolded are statistically significant

### Mentoring

Our study shows that more MD/PhD respondents (60.5%) and MD RI (50%) than MD respondents (35.6%) view mentorship as “very important” to their training so far (*p* < 0.001). A larger percentage of MD/PhD respondents (89.5%) and MD-RI (74.4%) than of MD respondents (67.4%) indicate that they currently have a mentor who has helped them progress toward and/or achieve their career goals (*p* < 0.001). Consistent with this, more MD (32.6%) than MD-RI (25.6%) have indicated that they were not able to identify a mentor who has helped them progress in their career goals (*p* < 0.0001), with MD/PhD (10.5%) having the lowest percent not being able to identify a mentor. There is no significant difference by MD or MD/PhD status in the levels of importance given to “talents/accomplishments” or “connections/networking” when recruiting applicants for jobs and/or positions in science and medicine. However, there is a trend toward MD/PhD (40.8%) and MD-RI (33.7%) giving talents/accomplishments a great deal of importance over MD (30.2%) students. Further, there is a trend of MD/PhD (33.3%) and MD-RI (32.8%) giving a great deal of importance to connections/networking over MD (28.3%) students.

### The influence of various factors on pursuing careers in academic medicine

Variables of interest for the logistic regression included those that differed significantly between the three groups in the prior tables. After removing variables with insufficient variance and those without significant bivariate relationships with the outcome variable (intention to go into academic medicine), 16 variables remained. Variables ultimately included in the model, in addition to MD/PhD status, female (as compared to male), White (as compared to all other categories) and Hispanic (as compared to non-Hispanic) included: paying for medical school primarily through loans; private practice as the desired career sector; 3 of the 7 potential career intentions (education, clinical research, and translational research); 2 of the residency areas (internal medicine and surgery); predicting eldercare after residency as a responsibility; predicting balancing clinical, research, and education as a career obstacle; desiring a 50/50 split between research and clinical duties; opportunities for community service as an important career factor; and whether respondents indicate being able to identify a mentor who has helped them progress toward and/or achieve career goals.

Controlling for the other variables, logistic regression analyses show that the MD/PhD candidate cohort was associated with a significantly increased likelihood of pursuing academic medicine when compared to their MD candidate cohort (330.2% increase; *p* < .05). Unexpectedly, the MD-RI candidate cohort was associated with a decreased likelihood of pursuing academic medicine compared to the MD candidate cohort (59.2% decrease; *p* < .05). Being female related to an increased likelihood of pursing academic medicine (74.4% increase; *p* < .05), but only in the model comparing MD-RI to MD. Likewise, being Hispanic related to a decreased likelihood of pursing academic medicine (62.8% decrease; *p* < .05), but only in the MD/PhD and MD cohorts. In all three models, four variables relate to an increased likelihood of pursuing academic medicine (*p* < .05): intending to focus on education, intending to focus on translational research, planning to specialize in internal medicine, and predicting eldercare responsibilities after residency. Another five variables related to increases in the likelihood of pursing academic medicine in 2 of the 3 models (*p* < .05): intending to focus on clinical research; specializing in surgery; predicting balancing clinical, research, and education duties as a career obstacle; desiring a 50/50 split between research and clinical time; and, being able to identify a mentor who has helped progression toward and/or achievement of career goals. Indicating that one paid for medical school primarily through loans related to a decreased likelihood of going into academic medicine for the MD/PhD and MD-RI comparison (86.8% decrease; (*p* < .05), but an increased likelihood for the MD/PhD and MD comparison (105.0% increase; (*p* < .05). Indicating plans to go into the private sector related to a decreased likelihood of pursing academic medicine (70.9% decrease, *p* < .05), but only in the MD/PhD and MD model. Lastly, indicating that opportunities for community service is a top three factor in selecting a career related to a decreased likelihood of pursing academic medicine (46.0% decrease, *p* < .05), but only in the MD-RI and MD model (Table 5).

**Table 5:** Logistic Regression on Career Plan after Residency: Academia

	Effects of degree focus on various factors related to academic career aspirations, AOR (95% CI)*		
Factor	MD/PhD vs. MD-Research *n* = 383	MD/PhD vs. MD-Non research *n* = 781	MD-Research vs. MD-Non research *n* = 740
MD/PhD	0.691 (0.145–3.286)	**4.302 (1.389–13.326)**	n/a
MD-Research Focus	1.000 (reference)	n/a	**0.408 (0.170–0.977)**
MD-Non Research	n/a	1.000 (reference)	1.000 (reference)
Female^a^	0.807 (0.274–2.373)	1.691 (1.019–2.808)	**1.744 (1.073–2.837)**
White^b^	2.762 (0.895–8.529)	1.632 (0.981–2.713)	1.373 (0.841–2.241)
Hispanic^c^	2.069 (0.196–21.790)	**0.372 (0.162–0.854)**	0.472 (0.216–1.030)
Paid primarily through loans	**0.132 (0.028–0.634)**	**2.050 (1.119–3.754)**	1.610 (0.922–2.813)
Sector: private practice	**0.291 (0.086–0.984)**	0.632 (0.358–1.117)	0.664 (0.387–1.139)
Intention: Education	**6.335 (1.888–21.254)**	**6.812 (4.094–11.334)**	**7.347 (4.535–11.904)**
Intend: Clinical Research	3.035 (0.901–10.224)	**3.281 (1.933–5.567)**	**2.911 (1.774–4.774)**
Intend: Translational Research	**6.055 (1.598–22.941)**	**2.165 (1.031–4.544)**	**2.195 (1.040–4.631)**
Spec: Internal Medicine	**5.021 (1.371–18.3920)**	**2.386 (1.388–4.104)**	**2.058 (1.221–3.473)**
Spec: Surgery	**2.925 (0.140–61.049)**	**2.664(1.053–6.739)**	2.338 (0.984–5.555)
Predict eldercare after res	**12.476 (2.653–58.907)**	**2.710 (1.641–4.475)**	**2.430 (1.490–3.963)**
Obs: Balance clin, res, ed	2.684 (0.892–8.073)	**4.401 (2.400–8.070)**	**3.749 (2.114–6.648)**
Desire 50:50 research:clinical	**4.691 (1.319–16.686)**	1.372 (0.243–7.760)	**7.695 (2.053–28.839)**
Career Factor: Community Svc	0.515 (0.131–2.029)	0.593 (0.336–1.047)	**0.540 (0.316–0.923)**
Can identify mentor	**3.204 (1.033–9.936)**	1.438 (0.585–2.411)	**1.836 (1.123–3.000)**

* Bolded values are statistically significant

^a^ Compared to male

^b^ Compared to self-report non-White

^c^ Compared to self-report non-Hispanic

## Discussion

Despite increasing knowledge of the eventual career paths of MD and MD/PhD graduates [4, 6, 30, 37, 38], relatively little has been done to assess the factors that influence the career choices of physician-scientists at the predoctoral candidate stage, particularly MD candidates interested in a career incorporating substantial research. Knowledge of the challenges and characteristics of early-stage trainees is critical to fostering interest in academic and research careers and supporting future physician-scientists with such interests. To our knowledge, our study provides one of the first comprehensive assessments of the aspirations, concerns, and perceptions of MD/PhD candidates, MD candidates interested in research-intensive careers (MD-RI), and MD candidates with little or no interest in research (MD) at the earliest stages of training. Our findings shed important light on a critical phase of physician-scientist training and may enable earlier and more accurate identification of trainees best suited for a physician-scientist career as well as identify the needs which, if unmet, may influence trainees to drop out of the career path entirely.

### Current and future obstacles

Attrition is an important problem facing physician-scientists at all career stages, and studies of MD/PhD programs have estimated attrition rates to be between a tenth [6] and a quarter of all matriculants [39]. Recognizing this, multiple investigations have attempted to quantify the importance of educational debt, MCAT scores, gender, race/ethnicity, and enrollment in an MSTP-funded MD/PhD program on likelihood of successful graduation [36, 38, 39]. Such studies have provided important insights, although the precise correlation of certain factors (gender and race/ethnicity in particular) with graduation remains debated. Unfortunately, attrition rates have yet to decline, suggesting that the complex issues facing trainee physician-scientists have yet to be fully defined.

Just over a decade ago, Ahn and colleagues surveyed a number of MD/PhD programs [35] and found troubling evidence that about a quarter of students had seriously considered leaving their program, although that study did not attempt to fully ascertain why. Our results may provide the first explanations for that high dissatisfaction, revealing that many of the same issues facing physician-scientist faculty are also felt by predoctoral trainees. In particular, demands outside of work (e.g. childcare, eldercare [25]) and cultural/environmental challenges (e.g. lack of professional advancement opportunities [23, 27]) that faculty struggle to overcome are precisely those that MD/PhD and MD-RI trainees report experiencing or expecting to experience at higher levels than their MD peers. If predoctoral candidates are already concerned about these issues, attempting to address them at the postdoctoral or faculty stage may be too little, too late. The recommendations of the recent Physician-Scientist Workforce report [20] (e.g. development of a pathway to independence award) provide a roadmap for future studies and interventions, while several early-career programs that have been successful in implementing good career development strategies [5, 24, 28–31] represent examples to which other academic leaders can look.

Intriguingly, one of the strongest similarities between our data and surveys of junior faculty lies in challenges surrounding raising a family that both groups experience or expect to experience. While MD/PhDs and MD-RIs differ in that these issues appear to be more immediate for MD/PhDs (i.e. many already are married and have children), the similarity is striking, and the differences likely arise from the increased length of training MD/PhDs undergo, allowing for more time to marry and start families prior to residency training. MD/PhD program directors and medical school leaders should make efforts to help support good family-work balance early in training, perhaps by specific accommodations such as flexible scheduling of required coursework or permitting greater flexibility in time to completion of degree or other training program (e.g. residency). This would allow trainees who need to take more time, such as to stay home with a new child, the opportunity to do so and later return to the training pathway. Trainees might also be assisted by the direct provision of child care by their academic institutions, an expensive prospect for the institution but likely no less expensive than the years and dollars invested in a trainee who leaves academic medicine for a more flexible career because of a lack of good childcare. In a more general way, connection of students with mentors who have been successful in raising families with an active career may prove an effective means to relay successful strategies for balancing the competing demands of work and family. With the proportion of US households consisting of two working spouses nearing 50% in 2015 [49], the old way of relying on one spouse to shoulder the burden is no longer an option. Indeed, data from the National Residency Match Program show that couples matching together have hit all-time highs in both of the last two years [50, 51]. Programs which do not take significant steps to accommodate trainee (and faculty) desires for work-life balance risk losing promising students who cannot see a future for themselves and their families in academia.

### Demographics

Although our sample is confined to 5 medical schools, demographics represents one important area in which our results confirm those of many others [17, 23, 36, 38, 44], namely that there are few under-represented minorities (URMs) among the physician-scientist cohort and that women are underrepresented amongst MD/PhDs as compared to MDs. However, our results reveal an interesting contrast. The percentage of Hispanics is nearly four-fold higher for MD-RI candidates than it is for MD/PhDs. What has discouraged research-inclined Hispanics from pursuing MD/PhD programs as this is seen at these institutions as well as in national trends? An important follow-up to our study that might address these questions would be a survey of Hispanic physician-scientists to ascertain how they became interested in the career and to identify specific obstacles they have encountered in the hope of providing solutions for current trainees.

However, despite the relatively higher proportion of Hispanic MD-RIs, URM status is a strong identifying factor for not being an MD/PhD or MD-RI candidate. Finances may provide one answer. While MD/PhDs report overwhelmingly that they are fully-funded by their programs, MD-RI and MD candidates express concerns regarding loans, and MD-RI candidates are uniquely concerned about under-compensation. Populations traditionally under-represented in academic medicine have been found to be financially disadvantaged as well [52], and this could lead to significantly higher educational debt burden prior to entering medical school. Facing reduced compensation as a physician-scientist when compared to their clinically-focused peers [22, 26], such candidates may be discouraged from pursuing the training path of a physician-scientist even when funded programs (i.e. MD/PhD) are available. A potential solution would be to adapt the existing NIH Loan Repayment Program to predoctoral candidates, allowing students with high debt burden to receive loan repayment in exchange for a commitment to research following graduation. Such a program would promote entry into MD/PhD programs and support those students who solely want to pursue the MD but also plan to undertake substantial research efforts during their professional careers.

An interesting aspect of our demographic data concerns the degrees held by the parents of trainees considering physician-scientist career. A parental MD, DO, or DVM was strongly associated with MD/PhD or MD-RI status, although a PhD had no particular association. This association suggests that early exposure to a medical career and medical science may inspire trainees to pursue research careers, and provides evidence that early exposure to the physician-scientist career path could be a means to encourage more trainees to consider research. It also provides some evidence for the deficit of under-represented minority students in either MD/PhD or MD-RI trainee cohorts. Under-represented minorities are a small fraction of the physician-scientist workforce and, indeed, the overall physician workforce [53], and the lack of exposure to parental mentors with advanced medical degrees could underlie some of the ensuing absence of minority trainees.

### Career plans

Previous studies of MD/PhD training programs suggested that a significant portion of MD/PhD graduates conducted basic (57%) and translational (41%) research [6], and our survey of candidate career intentions concurs, with a majority of MD/PhD candidates intent on pursuing one or both of these fields. Interestingly, very few MD candidates expressed interest in translational research, although many were intent on a career in clinical research, a difference that may arise from an overall preference against laboratory science (demonstrated by the low proportion aspiring to do basic research). Historically, MDs have made important basic science and translational research contributions, but both the proportion of basic science faculty who hold an MD or MD/PhD [54] and the absolute number of MD/PhD program alumni with primary appointments in basic science departments [6] has been declining for decades. Exploration of the specific factors turning otherwise research-inclined MD candidates away from these fields is urgently needed.

Our results also confirm the growing trend of physician-scientist trainees to pursue residency training in nontraditional fields [34, 35, 37]. While fields such as internal medicine and pathology attract the aspirations of the majority of physician-scientist trainees, nontraditional fields like radiology and radiation oncology are also seeing higher interest. Interestingly, this interest is consistent between MD/PhD and MD-RI candidates, perhaps indicative of broader shifts across the physician-scientist workplace. However, there are several important differences. While a sizeable minority of all physician-scientist trainees is confident that a research-intensive career in an acute care specialty is feasible, MD-RI candidates alone have a sizeable minority interested in surgical careers. Growing interest in the surgeon-scientist pathway has been noted previously [33], but contrasts with the relative lack of interest in our MD/PhD cohort. It is possible that such candidates have been discouraged by a lack of mentorship, as these fields have fewer role models to look to, and promoters of the surgeon-scientist pathway would do well to make such mentors available to MD/PhD programs and to provide specific career development assistance and advice to interested students.

### Future applications

Fostering interest in research amongst prospective physicians is a constant challenge, but an important one given the significant contributions physician-scientists have made to improving human health. One potential application of our survey data is as an assessment aid for medical schools. Directors of medical student research experiences at medical schools nationwide could assess their MD students to identify those who are most likely to pursue careers as physician-scientists early in their careers. Such early identification may be a key to fostering interest in the physician-scientist career path, as our data on the association of advanced degrees of parents to research career intentions suggests. Schools could assign research mentors to those students and provide intensive research experiences to enhance their training. Those MD/PhD programs that sometimes or exclusively solicit applications from accepted MD candidates could easily identify the most promising candidates. So-called “year-out” research programs, such as those offered by many specialty societies and the NIH, could target students identified by such analysis, not only attracting the best students to their programs but also enhancing the research training of those students and perhaps increasing the likelihood of those students pursuing careers in academic medicine, as one small effort to target residents has shown promise to do [31]. Furthermore, our survey data could provide an aid for MD/PhD programs in identifying outreach targets at the undergraduate level. Program directors face some challenges in identifying undergraduates with a full long-term commitment to a physician-scientist career, and application of our survey data to the design of an assessment tool for undergraduates may aid in eventually identifying undergraduates who might make promising MD/PhD candidates. We provide one example of such a model in the form of a support-vector machine (SVM), which was able to classify MD/PhD candidates, MD-RI candidates, and MD candidates with a high degree of accuracy (Additional file 1: Figure S1 and S2) based on their responses to questions like residency of interest, career intention and mother’s education/degrees (Additional file 1: Table S1). The type of modeling may be able to provide predictive factors that can help MD/PhD program directors identify applicants who are most likely to be committed to a physician scientist career pathway with strong research intentions.

### Potential weaknesses

A major limitation of our data concerns the generalizability of the results. We surveyed five schools with active Clinical Translational Sciences Awards (CTSA) and MSTPs from the Midwest and Northeast, only a subset of all 46 funded MSTPs [55], although it is likely that trainees attending MSTP- and CTSA-funded institutions share a similar level of commitment to research and the physician-scientist career. However, our surveyed institutions do not fully capture the population of trainees at MSTP-funded institutions. Furthermore, our data may not fully represent the spectrum of aspirations and expectations of trainees at the over 100 MD/PhD programs [56] or 158 schools of medicine [57] nationwide. Broader sampling of trainees nationally and across institutions is needed and we are currently implementing a national broader study of trainees. This national survey will assess a representative sample of MD and MD/PhD trainees across the country to explore the full breadth of aspirations and attitudes in this population. The present study represents an important building block for that national survey, identifying important questions and characteristics to target in the broader pool of trainees.

An important concern is that of response rate, with approximately 40% of eligible trainees responding to our survey. This necessarily raises the question of whether those who did not respond did so in a random manner or because of some underlying common quality (for example, that they were not interested in research). However, our response rate is not unusual in the context of prior surveys. Ahn (Ahn 2004) and Watt (Watt 2005) achieved nearly 60% response rates, but these were limited to a single institution. Other surveys of MD students saw response rates in the low to mid-40% range, similar to ours. Other surveys of MD-PhD graduates have achieved “response” rates from 70% to nearly 100%, but these utilized either surveys of MD-PhD programs themselves [6] (Brass 2010) or analysis of AAMC graduate data [36–38]. While providing important insights into student choices and obstacles, such studies necessarily are more distant from the direct opinions of the trainees themselves.

A further concern arises from potential biases of our respondents. Focusing our survey on science, research, and inclusion of these in a career may have introduced bias in favor of options that suggested a research career, perhaps by prompting subjects to consider research and their career more closely than they otherwise would have. Additionally, the nature of our survey may have tended to recruit trainees with pre-existing interest in research while providing less incentive for those intent on clinical careers. Biases in this area may be more apparent for MD and MD-RI candidates than for MD/PhD candidates, as MD/PhD candidates would be expected to possess stronger interest in a research career overall.

Finally, an important consideration when evaluating our survey data is the necessarily-prospective nature of the questions and responses. Our survey asked participants to predict career outcomes, a prospect necessarily including some degree of uncertainty. However, it is important to assess the attitudes and expectations of students in the process of training, for that is the time when key decisions are being made. Future studies could re-assess our survey population to assess their actual career choices, comparing them to expectations and predictions in the present study.

## Conclusions

This study provides the first broad assessment of the factors driving the decision to embark on a career as a physician-scientist at the candidate level. We have identified experienced and predicted obstacles to academic career success for MD and MD/PhD candidates, including issues of work-life balance and future career development opportunities. We have shown that many of the same issues affecting MD/PhDs, and attitudes held by them, are also experienced by MD-RIs and that these differ from MDs with little or no research interest. Our data provide further evidence of the low diversity of the physician-scientist workforce and highlight and reinforce emerging trends in physician-scientist career choices, including an increasing number of candidates aspiring to nontraditional fields of practice. Of note, this study provides a snapshot of current trainee goals, important factors and obstacles in deciding their careers. It will be important to be able to follow such a cohort from baseline to several time points out along their careers to assess what factors influenced their ability to remain in or leave academia and/or research careers.

These findings help provide insights for institutional planning and infrastructure development that could help seal the leaky pipeline of physician-scientist training and ensure that the important contributions of physician-scientists continue to increase our understanding of disease mechanisms and thereby improve human health.

## Additional file

Additional file 1
**Figure S1**. ROC curves generated via SVM modeling using unsupervised responses to survey questions that helped distinguish MD vs MD-RI vs MD PhD trainees. **Figure S2**. Support vector machine modeling of MD vs MD-RI vs MD PhD trainees. **Table S1**. Responses to questions that more most predictive in modeling and classifying the MD vs MDRI vs MD PhD cohorts. (DOCX 222 kb)

## Abbreviations

AAMC: Association of American Medical Colleges
APSA: American Physician Scientists Association
CDAs: Career development awards
CTSA: Clinical and translational science awards
G1: Graduate School, Year 1
G2: Graduate School, Year 2
G3: Graduate School, Year 3
G4: Graduate School, Year 4
G5: Graduate School, Year 5
M1: Medical School, Year 1
M2: Medical School, Year 2
M3: Medical School, Year 3
M4: Medical School, Year 4
MD-RI: MD candidates with research-intense career intentions
MSTPs: Medical scientist training programs
NIH: National Institutes of Health
SVM: Support-Vector Machine
URMs: Under-Represented Minorities
