# Supplementary material for: Exploring intentions of physician-scientist trainees: factors influencing MD and MD/PhD interest in research careers
Source: BMC Med Educ. 2017 Jul 11;17:115. doi: 10.1186/s12909-017-0954-8 (PMC5505137; doi:10.1186/s12909-017-0954-8)
Supplement: Additional file 1 — Figure S1. ROC curves generated via SVM modeling using unsupervised responses to survey questions that helped distinguish MD vs MD-RI vs MD PhD trainees. Figure S2. Support vector machine modeling of MD vs MD-RI vs MD PhD trainees. Table S1. Responses to questions that more most predictive in modeling and classifying the MD vs MDRI vs MD PhD cohorts. (DOCX 222 kb) [file 12909_2017_954_MOESM1_ESM.docx]

Supplemental Methods and Discussion

We used advanced machine learning algorithms to establish the utility of our survey questionnaire for identifying clinicians likely to dedicate a substantial portion of their career to research pursuits. Machine learning techniques can allow us to deduce a research orientation score using user responses to the survey questionnaire. The potential application of such a score includes career counseling as well as candidate screening.

We divided the individuals in the survey into three categories a) MD candidates who plan to dedicate less than 50% of their time to research, b) MD candidates who plan to dedicate more than 50% of their time to research, and c) MD-PhD candidates. In order to understand what latent factors can be predictive of this choice, we eliminated survey responses directly querying the candidates about the proportion of their research commitment.

A one versus one support vector machine (SVM) based multiclass classification engine was constructed in Matlab using LIBSVM^1^ and custom code as previously described^2^. The SVM was trained to distinguish between the three categories of individuals based on their survey responses. This approach first trained three separate SVMs, one for distinguishing MDs vs. research intense MDs (MD-RI), one for distinguishing MDs vs MD-PhDs and one for distinguishing research intense MDs vs. MD-PhDs (Supplemental Figure 1). The final labeling was done by combining the scores generated by each of these classifiers (Supplemental Figure 2). Supplemental Table 1 lists the top five questions each classifier deemed pertinent to making the prediction.

Supplemental Figure 1. ROC curves using pertaining to classifiers for MD, MD-RI, and MD-PhD

**A)**


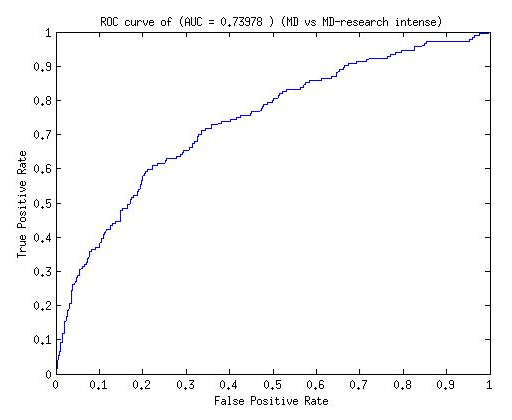


**B)**


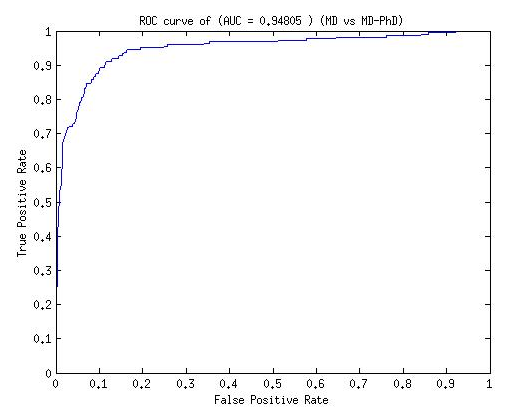


**C)**


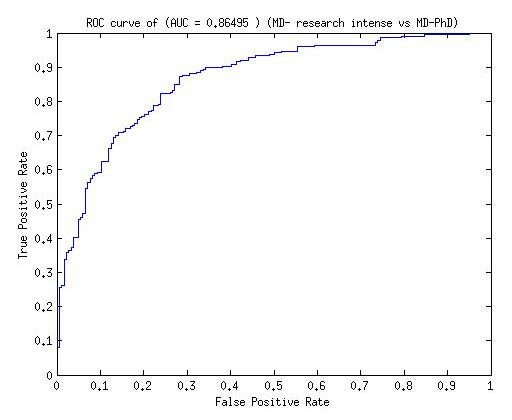


ROC curves using 10 fold cross validation pertaining to classifiers associated with (A) MD vs MD-RI (B) MD vs MD-PhD, and (C) MD-RI vs MD-PhD. The MD vs MD-PhD classification generates the strongest ROC curve, with an AUC of 0.94. The scores generated by applying this classifier to the MD-RI subgroup lie between the scores assigned by it to the MDs and the MD-PhDs (see Supplemental Figure 2).

Supplemental Figure 2. Support vector machine (SVM) achieves a high accuracy in distinguishing MD-research focused (MD-RI), MD non-research focused (MD), and MD/PhD trainees


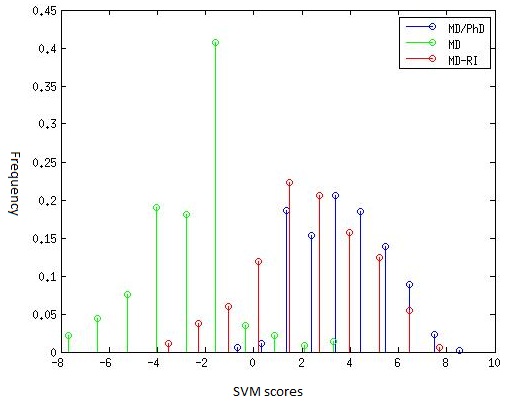


Stem plot of 10 fold cross validation-based support vector machine (SVM) scores. Stems of scores assigned to MD/PhD, MD-RI, and MD are plotted in blue, red, and green, respectively. The small region of overlap is the source of classification errors, as leave-one-out cross-validation accuracy is less than 100%. The y-axis represents percentage of respondents in each subgroup. The cross validation accuracy of distinguishing the three classes is 75%. A pure random guess would achieve 33% accuracy in the three class case.

Supplemental Table 1**.**  Questions most relevant to SVM predictions (top five for each sub-classifier)

| MD vs MD-RI | Top two residencies of interest  What areas of medicine is your father working in  What 3 factors are most important to you in selecting a career  Specify advanced degrees of your mother  What areas of medicine is your mother working in |
| --- | --- |
| MD vs MD-PhD | What are your career intentions  Specify advanced degrees of your mother  Top two residencies of interest  What areas of medicine is your mother working in  Non work-related responsibilities do you foresee DURING residency |
| MD-RI vs MD-PhD | Top two residencies of interest  What do you foresee as obstacles in career  What are your career intentions  What are your foreseeable non work-related responsibilities for you AFTER residency?  So far in your training, what have you encountered as a hindrance to your career advancement? |

Supplemental References

1. Chang C-C, Lin C-J. LIBSVM: A library for support vector machines. *ACM Trans Intell Syst Technol*. 2011;2(3):1-27.

2. Gaonkar B, Davatzikos C. Analytic estimation of statistical significance maps for support vector machine based multi-variate image analysis and classification. *NeuroImage*. 2013;78:270-283. doi:10.1016/j.neuroimage.2013.03.066.
